# Spatial Modelling of Aerial Survey Data Reveals an Important European Storm‐Petrel Hotspot and Its Underlying Drivers Within the North‐East Atlantic

**DOI:** 10.1002/ece3.71438

**Published:** 2025-06-30

**Authors:** Darren Wilkinson, Jamie Darby, Ashley Bennison, Hélder Araújo, Oriol Giralt Paradell, T. David Tierney, Emer Rogan, John L. Quinn, Mark Jessopp

**Affiliations:** ^1^ School of Biological, Earth and Environmental Sciences University College Cork Cork Ireland; ^2^ School of Biological Sciences University of Auckland Auckland New Zealand; ^3^ British Antarctic Survey Cambridge UK; ^4^ Biology Department & ECOMARE University of Aveiro Aveiro Portugal; ^5^ MaREI Centre for Energy, Climate and Marine Environmental Research Institute, University College Cork Cork Ireland; ^6^ National Parks and Wildlife Service Department of Housing, Local Government and Heritage Dublin Ireland

**Keywords:** Aerial survey, European storm‐petrel, North‐East Atlantic, population abundance, species distribution modelling

## Abstract

Determining the distribution and population size of marine species is crucial for conservation and management. However, for many species, the abundance and at sea distribution are poorly known because of their large geographic ranges, high mobility and cryptic breeding habits. This is especially true for small pelagic seabirds such as the European storm‐petrel. Large‐scale observer‐based aerial surveys were conducted over four summers in the North‐East Atlantic extending 200 nautical miles from the coast of Ireland. Species distribution models were produced using generalised additive models with a combination of static and dynamic environmental variables to assess the impact of survey altitude on storm‐petrel detectability, and to model their abundance and distribution. Reduced storm‐petrel detectability was identified at higher survey altitudes and rougher seas, and an at‐sea abundance of 154,044 (95% CI: 94,347–452,299) individuals was estimated. Our results reveal fine‐scale variation in the spatial distribution of storm‐petrels and highlight the unsuitability of foraging radius distribution models for such species. Storm‐petrels were found to avoid coastal areas, which we speculate is linked to the avoidance of large coastal avian predators during the day. Although the continental shelf edge was highlighted as a significant feature in the distribution of this pelagic species, a more prominent hotspot was identified in neritic areas, 20–40 km off the south and south‐west coasts of Ireland in a region highly influenced by shelf fronts, coastal currents, upwellings and eddies in the summer months. The identified hotspot has global significance since Ireland holds more than 20% of the entire European storm‐petrel breeding population.

## Graphical abstract

Determining the distribution and population size of marine species, especially small pelagic seabirds, such as the European storm‐petrel, is crucial for conservation but is challenging due to their wide ranges, high mobility and cryptic breeding habits. Species distribution modelling of large‐scale aerial survey data was conducted to assess the suitability of this method for monitoring storm‐petrels at sea and identified abundance hotspots 20–40 km from the coast in a region highly influenced by shelf fronts, coastal currents, upwellings and eddies. The identified hotspot has global significance since Ireland holds more than 20% of the entire European storm‐petrel breeding population.

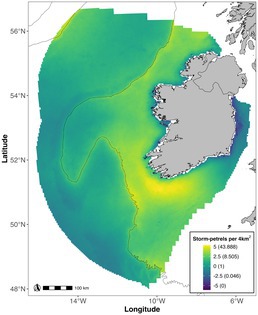

## 1 Introduction

Seabirds are one of the most threatened avian groups (Croxall et al. 2012; Dias et al. 2019) with monitored populations showing large‐scale declines globally in recent decades (Paleczny et al. 2015). Many threatened seabird species are subject to multiple pressures, including introduced mammalian predators, fisheries bycatch, climate change, marine pollution and overfishing (Dias et al. 2019). Seabirds are regarded as sentinels of the marine environment (Grémillet and Charmantier 2010; Sydeman et al. 2021), and population declines and shifts in species distribution are of great concern as they may signal broader ecosystem issues.

Understanding the at‐sea distribution of seabirds, particularly the high‐use areas, is essential for effective conservation and management (Croxall et al. 2012; Rodríguez et al. 2019). This is crucial in the context of the rapidly expanding offshore renewable energy sector, which is expected to continue growing in the coming years. For many species, collecting this data can be challenging due to their large geographic ranges, remote breeding habitats and high mobility. Rapid advancement of telemetry technology has greatly improved our knowledge of seabird at‐sea distribution and behaviour (Burger and Shaffer 2008). Nevertheless, telemetry studies typically neglect a substantial portion of the population (non‐breeders and those breeding in inaccessible locations) and focus only on a small sample of colonies across their entire range because of logistical and financial constraints (Ronconi et al. 2022), unless extensive collaboration is possible (e.g., Wakefield et al. 2013).

At‐sea visual surveys, conducted from boats (e.g., Tasker et al. 1984; Van der Meer and Leopold 1995; Kober et al. 2010) or aircraft (e.g., Maclean et al. 2006; Certain and Bretagnolle 2008; Merkel et al. 2019; Virgili et al. 2024) offer an alternative method of examining the abundance and distribution of seabirds. Compared to telemetry studies, at‐sea surveys have the advantages of establishing the presence, absence and density of the target species across the study area, recording sightings of breeding and non‐breeding individuals, and simultaneously gathering occurrence data for other seabird and megafauna species. Aerial surveys also collect data suitable for studying seabird population abundances (e.g., Dean et al. 2003; Bretagnolle et al. 2004; Camphuysen et al. 2004; Buckland et al. 2012; Winiarski et al. 2013; Pettex, David, et al. 2017; Pettex, Laran, et al. 2017; Merkel et al. 2019; Ford et al. 2021; Araújo et al. 2022), offering insights into long‐term trends and serving as an alternative method of monitoring seabird populations that lack sufficient data or are difficult to assess through other approaches. Although breeding adults of ground‐ and cliff‐nesting species are relatively simple to census through visual surveys at breeding colonies, burrow‐nesting seabirds are notably difficult to census due to their cryptic breeding habits, as many species exclusively return to colonies at night.

The growing accessibility of remotely sensed environmental data and at‐sea survey data has greatly improved our understanding of seabird habitat use over various spatial and temporal scales (Lascelles et al. 2012). Oceanographic and geospatial variables can be used as proxies for the marine habitats that seabird species use. Variables, such as chlorophyll *a* concentration, sea surface temperature (SST) and ocean productivity indices are frequently used as dynamic indicators of prey availability (e.g., Tremblay et al. 2009; Domalik et al. 2018; Serratosa et al. 2020) but they can have low predictive power (e.g., Kane et al. 2020). In contrast, several studies have found static variables relating to features of the marine environment (bathymetry and coastal/breeding colony proximity) to better reflect the at‐sea distribution of seabirds (e.g., Amorim et al. 2009; Nur et al. 2011). Species distribution models (SDMs) incorporating these dynamic and fixed variables have been widely used to study the relationships between species and their environment (Guisan and Zimmermann 2000) and allow for the prediction of a species' distribution and density over different spatio‐temporal scales. SDMs using observation data collected from at‐sea visual surveys in combination with physical and environmental variables have emerged as key methods for predicting the distribution and abundance of seabirds at sea (e.g., Virgili et al. 2017). Thus, they are valuable tools when making conservation decisions (Guisan et al. 2013; Krüger et al. 2017).

Although there are clear benefits of using aerial surveys for the study of seabird distribution and abundance, errors can be introduced into the data through imperfect detection of the target species by observers (Davis et al. 2022). Consequently, careful interpretation of the results is necessary. Factors such as aircraft altitude and speed, survey strip width and weather conditions can influence the detection of seabirds (Briggs et al. 1985; Camphuysen et al. 2004; Certain and Bretagnolle 2008). Detection issues can be mitigated to some extent through careful survey design and strict weather criteria for survey flights, though errors can still result from (1) counting mistakes in which observers under‐ or over‐report the true number of individuals in a transect, (2) species misidentification and (3) non‐detection in which individuals are present but not observed such as when diving species are under water (Davis et al. 2022). These issues are typically less of a concern for large, conspicuous species (Certain and Bretagnolle 2008) but aerial surveys have still been used successfully to study the abundance and distribution of more cryptic seabirds such as auks (e.g., Bretagnolle et al. 2004; Pettex, David, et al. 2017; Pettex, Laran, et al. 2017; Waggitt et al. 2020; Araújo et al. 2022). However, there is a considerable lack of knowledge on the detection accuracy and optimum design of aerial surveys for the smallest seabird species in our oceans (i.e., the storm‐petrels, Hydrobatidae and Oceanitidae).

Using species distribution modelling of aerial survey data, the aim of this study was to better understand the at‐sea distribution and abundance of one of the smallest seabirds globally, the European storm‐petrel ( *Hydrobates pelagicus* ), in an important region of their North‐East Atlantic range. The European storm‐petrel has a widespread distribution across Europe, with Ireland holding an estimated 20%–23% of the global breeding population (Burnell et al. 2023) and the breeding season typically spans from late May to early October (Scott 1970). SDMs have been constructed previously for this species using ship survey data (De la Cruz et al. 2023), whereas the European storm‐petrel has also featured in multi‐species studies using aerial survey data (Pettex, David, et al. 2017; Pettex, Laran, et al. 2017; Araújo et al. 2022; McGovern et al. 2022), or combinations of both ship and aerial surveys (Waggitt et al. 2020). To the best of our knowledge, this study represents the first detailed analysis to exclusively focus on European storm‐petrel distribution and abundance using aerial survey data. Over 4 years, aerial surveys were used to record sightings of marine megafauna in Ireland's coastal and offshore waters. Using this dataset, the goals of this study were (1) to assess the impact of survey altitude on the detectability of storm‐petrels; (2) to better understand the distribution of European storm‐petrels in an important area of their North‐East Atlantic range; and (3) to compare the abundance estimate generated from the model to published population estimates, determining whether aerial surveys are a suitable method for regular monitoring to detect population trends.

## 2 Material and Methods

### 2.1 Aerial Surveys and Data Collection

**FIGURE 1:**
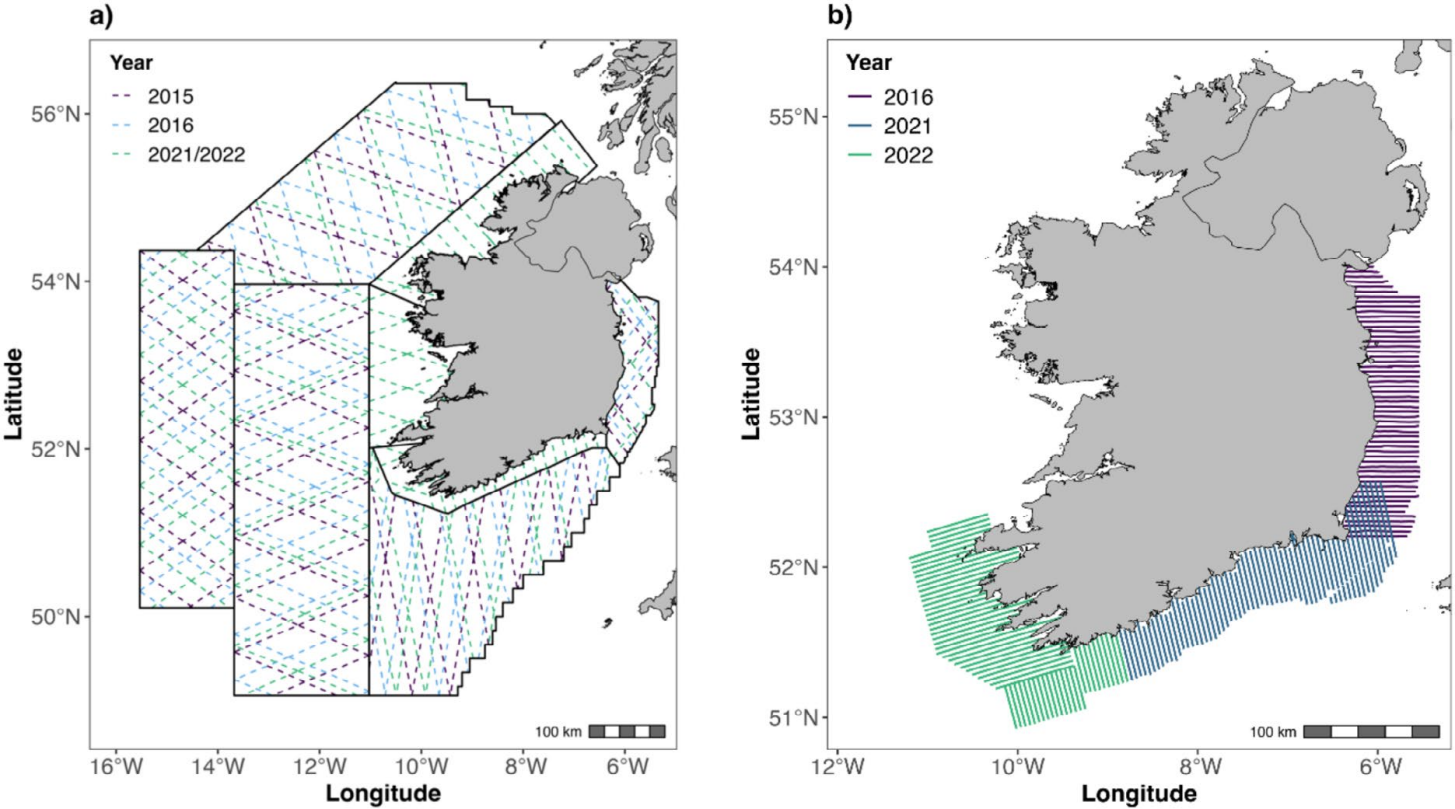
(a) Broad‐scale aerial survey transects in 2015 (purple dashed lines), 2016 (blue dashed lines), and 2021/2022 (green dashed lines) within the eight survey strata (black solid lines). (b) Fine‐scale aerial survey transects conducted in 2016 (purple), 2021 (blue), and 2022 (green).

The study area was the offshore and coastal waters of the Irish Exclusive Economic Zone (EEZ). This covers an area of approximately 450,000 km^2^ which is more than six times larger than Ireland's land area. A combination of broad‐ and fine‐scale aerial surveys were conducted by a team of trained observers in a twin‐engine, high wing aircraft equipped with bubble windows to provide an unobstructed view of the trackline directly below the aircraft. Surveys were conducted between May and September in 2015, 2016, 2021, and 2022 (Table S1) and followed a standard strip‐transect methodology (Buckland et al. 2001). Broad‐scale surveys divided the entire study area into strata and transects were designed within each stratum to ensure equal coverage probability (Figure 1a). Fine‐scale aerial surveys consisting of parallel transects, perpendicular to the coast to ensure that they covered the bathymetric gradient, and spaced approximately two nautical miles apart were conducted in the coastal waters of the Irish Sea in 2016, along the south coast in 2021, and the south‐west coast in 2022 (Figure 1b). Fine‐scale surveys were conducted to investigate the occurrence and abundance of megafauna using the shallow productive waters around the coast at finer spatial resolution. Flying speed for all surveys was 90–100 knots at an altitude of 183 m (600 ft) for broad‐scale surveys and 76 m (250 ft) for fine‐scale transects. All sightings of seabirds, including storm‐petrel species, within 200 m of the transect line on each side of the aircraft, and the group size, were recorded. Due to the similarity between storm‐petrel species, identification to the species‐level was not possible. Other storm‐petrel species including Leach's storm‐petrels (*H. leuchorus*) and Wilson's storm‐petrels ( *Oceanites oceanicus* ) are much less abundant in the region (Flood and Thomas 2007; Burnell et al. 2023) and are expected to account for a very small minority of the at‐sea observations of storm‐petrels in this area. Sightings of other marine megafauna species (e.g., cetaceans, sharks) were recorded using distance sampling methods and we assume this, and the presence of other seabird species or fishing vessels, did not impact the sightings of storm‐petrels. Beaufort sea state, cloud cover, glare and precipitation were logged at the start of each transect and whenever a noticeable change in conditions was observed. The desired weather and sea conditions for aerial surveys were generally defined as requiring surface wind speeds of Beaufort Force 3 or less, and with visibility of at least 1 km. At least 95% of the survey effort was conducted in Beaufort sea state 3 or less in all survey periods except summer 2015 (Table S1), during which 98.7% of the broad‐scale survey effort was performed in Beaufort sea state 4 or less. The aircraft's position was recorded every 2 s using the on‐board GPS connected to the data logging software (VOR in 2015, 2016, and 2021, and SAMMOA 2.1.0 in 2022). Audio backup was recorded using a Zoom H1n Dictaphone in 2015–2021 and an in‐built recording system in the SAMMOA software in 2022.

### 2.2 Data Handling

All analyses were completed using R v4.3.2 (R Core Team 2023). A spatial grid of 4 × 4 km resolution, comparable to the finest resolution of environmental variables used in this study, was created covering the entire study area resulting in 26,705 grid cells, and the total number of storm‐petrels recorded in each grid cell was calculated from the broad‐ and fine‐scale surveys. A primary assumption of the strip‐transect methodology is that 100% of storm‐petrels within the strip width are seen and accurately recorded. Although this has been demonstrated to be reasonable for aerial surveys of both highly conspicuous species, such as gannets ( *Morus bassanus* ) and cryptic auk species (Certain and Bretagnolle 2008), no analysis on species as small as storm‐petrels has been conducted to date, and this assumption is likely to lead to underestimates of the actual presence and abundance of storm‐petrels.

A total of 51,165 km of survey transects were included in the analysis, and after allowing for the 200 m strip width on either side of the plane but removing any parts where observers were off effort due to poor viewing conditions (e.g., low cloud or strong glare), this equated to 19,881 km^2^ of surface area (4.4% of the entire area of the Irish EEZ and 36% of 4 × 4 km grid cells) being surveyed over 4 years between 2015 and 2022. The area of the survey transects within each grid cell was calculated using the *sf* package (Pebesma 2018).

### 2.3 Environmental Variables

Environmental variables that were predicted to influence storm‐petrel at‐sea distribution were appended to the centre point of each grid cell. Distance to the coast, distance to the 500 m isobath (representing the continental shelf edge), and seabed depth of the centre point of each grid cell were calculated using the *marmap* package (Pante et al. 2023). Seabed depth was set to 0 m where a value above sea level was detected due to the grid cell centre point being located over land (e.g., islands). Chlorophyll *a* concentration (chl‐*a*; mg m^−3^) and SST (°C) were sourced from MoveBank's Env‐DATA service (Dodge et al. 2013), which uses NASA's MODIS satellite data. These data were sourced at 8‐day temporal and 4 km spatial resolution and appended using inverse‐distance‐weighted interpolation. Daily sea surface salinity (SSS; ‰) was acquired from the Copernicus Marine Service Ocean Products database (marine.copernicus.eu) and the 8‐day mean was calculated for each grid cell to match the temporal resolution of the other environmental variables. Seabed slope and gradients of chl‐*a*, SST, and SSS were estimated using the ‘terrain’ function in the *raster* package (Hijmans 2023). Gradients of environmental variables such as SST have been used in distribution modelling, with high values identifying the potential presence of oceanographic features such as fronts (Péron et al. 2018; Goh et al. 2024). Storm‐petrel colony locations (Figure S1) and population estimates were obtained from Burnell et al. (2023). A colony proximity score was calculated for each grid cell by considering distance to the breeding colonies and the population of each colony. This was done as grid cells closer to large colonies have the potential to be visited by more storm‐petrels than cells close to smaller colonies. For each colony, a 4 × 4 km resolution raster was created, calculating the shortest distance, avoiding land, from each cell centre to the colony using the *raster* package (Hijmans 2023). The number of breeding pairs was divided by distance^2^ for each cell, and these values were summed across all colony rasters to produce a single value for each grid cell (Wakefield et al. 2011). The Julian day on which each grid cell was surveyed was determined, and the mean sea state of each surveyed grid cell was calculated using the observers' records for the section of the transect that overlapped with the grid cell. Variables were transformed using a Box‐Cox transformation to improve normality where required (Table S2).

### 2.4 Impact of Survey Altitude on Monitoring Storm‐Petrels

Generalised additive models (GAMs; Hastie and Tibshirani 1990) were used to examine factors affecting storm‐petrel sightings using the ‘gam’ function in the *mgcv* package (Wood 2011). Due to the small size of storm‐petrels, it was hypothesised that storm‐petrels would be detected more frequently in the fine‐scale survey transects compared to the broad‐scale surveys, when all other factors are equal, as the lower altitude would make spotting storm‐petrels easier. To test this, storm‐petrel abundance was modelled as a function of a combination of fixed and dynamic covariates using a negative binomial distribution with a log link function. As the lower altitude fine‐scale surveys were conducted in coastal waters and the broad‐scale surveys largely covered areas further offshore, only the 838 grid cells that were surveyed by both broad‐ and fine‐scale transects were retained for the analysis to prevent the effect of survey altitude being masked by the spatial variables. Survey effort was included in the model as the logarithm of the area of each grid cell surveyed and incorporated as an offset term. X and Y coordinates were included as a bi‐dimensional spline with shrinkage to help account for spatial autocorrelation while year was entered as a factor. A factor‐smooth interaction between mean sea state and survey altitude (binomial term “high” and “low” for broad‐ and fine‐scale surveys, respectively) was included, as any potential effects of survey altitude on the detectability of storm‐petrels could be exacerbated at higher sea states. Survey altitude was also included in the model as a parametric term due to the centring constraints applied in the factor‐smooth interaction. All other variables were fitted with a maximum of five knots and using penalised thin‐plate regression splines with shrinkage to return the simplest effective response curve and avoid overfitting. The gamma parameter was set to 1.2 to reduce complexity further by increasing the null space penalty (Wood 2003). Covariates highly correlated (> 0.8) were identified using the ‘concurvity’ function in the *mgcv* package (Wood 2011). Models containing all combinations of these correlated covariates were produced, with the model possessing the lowest Akaike Information Criterion (AIC) being considered best, regardless of whether some correlated terms were retained as partial effects may be expressed by related variables (Morrissey and Ruxton 2018). Model selection was semi‐automated by applying an additional penalty to the model terms whose smoothing parameter was approaching zero. This feature within mgcv's ‘gam’ function regresses the effect of non‐contributing covariates to zero, effectively removing them from the model. All model checks were conducted using the *DHARMa* package (Hartig 2022) and the model goodness of fit was described using deviance explained.

### 2.5 Distribution and Abundance Modelling

A GAM approach, using the *mgcv* package (Wood 2011), was implemented to examine the distribution and abundance of storm‐petrels across the study area. The number of storm‐petrels per grid cell was modelled using a negative binomial distribution with a log link function and a dataset consisting of every grid cell surveyed during each aerial survey period. The logarithm of survey effort was included as an offset term, and a factor‐smooth interaction between mean sea state and survey altitude was incorporated to account for the potential decline in detectability at higher sea states and altitudes. Survey altitude was additionally included as a parametric term. X and Y coordinates were included as a bi‐dimensional spline with shrinkage to account for spatial autocorrelation, and year was entered as a random effect, as opposed to a factor, due to the imbalanced sampling across years and the study's focus on general distribution patterns rather than year‐specific effects. Distance to the coast, distance to the continental shelf edge, colony proximity score, seabed depth, seabed slope, chl‐*a*, SST, SSS, chl‐*a* gradient, SST gradient, SSS gradient and Julian day were included using penalised thin‐plate regression splines with shrinkage. A maximum of five knots was set for each covariate, but this was increased for terms where potential underfitting was identified. Once again, all combinations of correlated covariates were modelled with the model possessing the lowest AIC being considered best, regardless of whether any correlated terms remained to avoid masking partial effects of related variables (Morrissey and Ruxton 2018). The method by which *mgcv* fits GAMs, using backfitting to estimate covariates, also helps mitigate the negative impacts of multicollinearity (Wood 2008). The gamma parameter was set to 1.2 to reduce spline complexity by increasing the null penalty (Wood 2003) and the smoothing parameters were allowed to regress to zero if the model terms had no effect. Model suitability was checked with simulated residuals using the *DHARMa* package (Hartig 2022) and the model goodness of fit was described using deviance explained.

### 2.6 Validation and Prediction

To validate the model, the dataset was randomly split into training data (80%) and test data (20%) for 100 iterations. Using each training dataset, the model was run and used to predict storm‐petrel abundance for the accompanying test dataset. Suitability of the model was examined by comparing the total predicted abundance with the actual abundance for each of the test datasets.

Once validated, the final model was used to predict the distribution and abundance of storm‐petrels in the entire study area using the ‘predict.gam’ function within the *mgcv* package (Wood 2011). A new 4 × 4 km spatial grid covering the Irish EEZ was constructed for the prediction. Distance to the coast, distance to the continental shelf edge, colony proximity score, seabed depth and seabed slope were obtained using the methods outlined previously (Figure S2). The median Julian day (195) was used for the predictions while the Beaufort sea state was set to zero and survey altitude to “low”, to reflect the calm sea conditions and survey design when detection of storm‐petrels is optimal (see Section 3). Chl‐*a* and SST data were obtained at 8‐day and 4 km resolution for the median Julian day for every year between 2015 and 2022 from NASA's Ocean Biology Processing Group service (oceandata.sci.gsfc.nasa.gov/l3/). The 8‐day mean for SSS at the median Julian day was calculated from daily data downloaded from the Copernicus Marine Service Ocean Product database (marine.copernicus.eu). The mean values of chl‐*a*, SST and SSS over this timescale were appended to the grid cell centre points and the gradients were calculated using the ‘terrain’ function from the *raster* package (Hijmans 2023; Figure S2). Variables that were transformed prior to model fitting were subjected to a Box‐Cox transformation, using the same λ value as in the initial transformation. Total storm‐petrel abundance for the entire study area was obtained by summing the predicted abundance of each grid cell and rounding to the nearest whole number. Other than the inclusion of Beaufort sea state and survey altitude in the model, the abundance estimate was not corrected for potential detection bias and therefore is likely an underestimate of the true at‐sea abundance of storm‐petrels in the study area. Consequently, the abundance estimate should be interpreted as a minimum value. The coefficient of variation for each grid cell was calculated and the 95% confidence interval (CI) for the abundance prediction was produced using a percentile bootstrapping procedure with 1000 iterations and performed using the *boot* package (Canty and Ripley 2024). A strong positive skew in the sampling distribution used for bootstrapping, driven by outliers, can distort confidence interval estimates (Singh 1998). To mitigate this, winsorisation was applied prior to bootstrapping by capping the highest storm‐petrel counts at the upper 97.5th percentile value. This approach reduces the influence of extreme values, ensuring that the bootstrapped estimates are more stable (Wilcox 2012).

## 3 Results

There were 2742 storm‐petrel sightings totalling 4487 individuals (Table S3). Most storm‐petrels were detected over the continental shelf, with some sightings in deeper waters beyond the shelf edge (Figure S3). Sightings of individual storm‐petrels were most common (80.5% of sighting events), and petrel pairs accounted for 10.7% of sightings.

### 3.1 Effect of Survey Altitude on Storm‐Petrel Sightings

The final negative binomial GAM, which explained 53.1% of deviance, excluded distance to the coast and SSS due to multicollinearity. In addition, the effects of distance to the continental shelf edge, colony proximity score, SST, chl‐*a* and chl‐*a* gradient were reduced to zero, whereas seabed slope and SSS gradient were non‐significant (Table S4). The survey altitude factor was significant while there was also a significant effect of sea state at the higher altitude broad‐scale survey data, and the interaction was approaching significance for the fine‐scale data (Table S4). This suggests that storm‐petrel detectability decreases at higher survey altitudes and under rougher sea conditions, particularly when both factors are combined.

### 3.2 Distribution and Abundance of Storm‐Petrels

The test datasets revealed moderate‐good prediction accuracy when compared to the observed storm‐petrel abundance. Residuals were normally distributed (Figure S4), with 29% of the test abundance predictions falling within 5% of the observed abundance and approximately two‐thirds within 10% of observed abundance (Figure S4). Overall, the validation models tended to underestimate the actual abundance, suggesting that the total abundance estimate generated by the final model is likely a conservative approximation of the true storm‐petrel at‐sea abundance.

**FIGURE 2:**
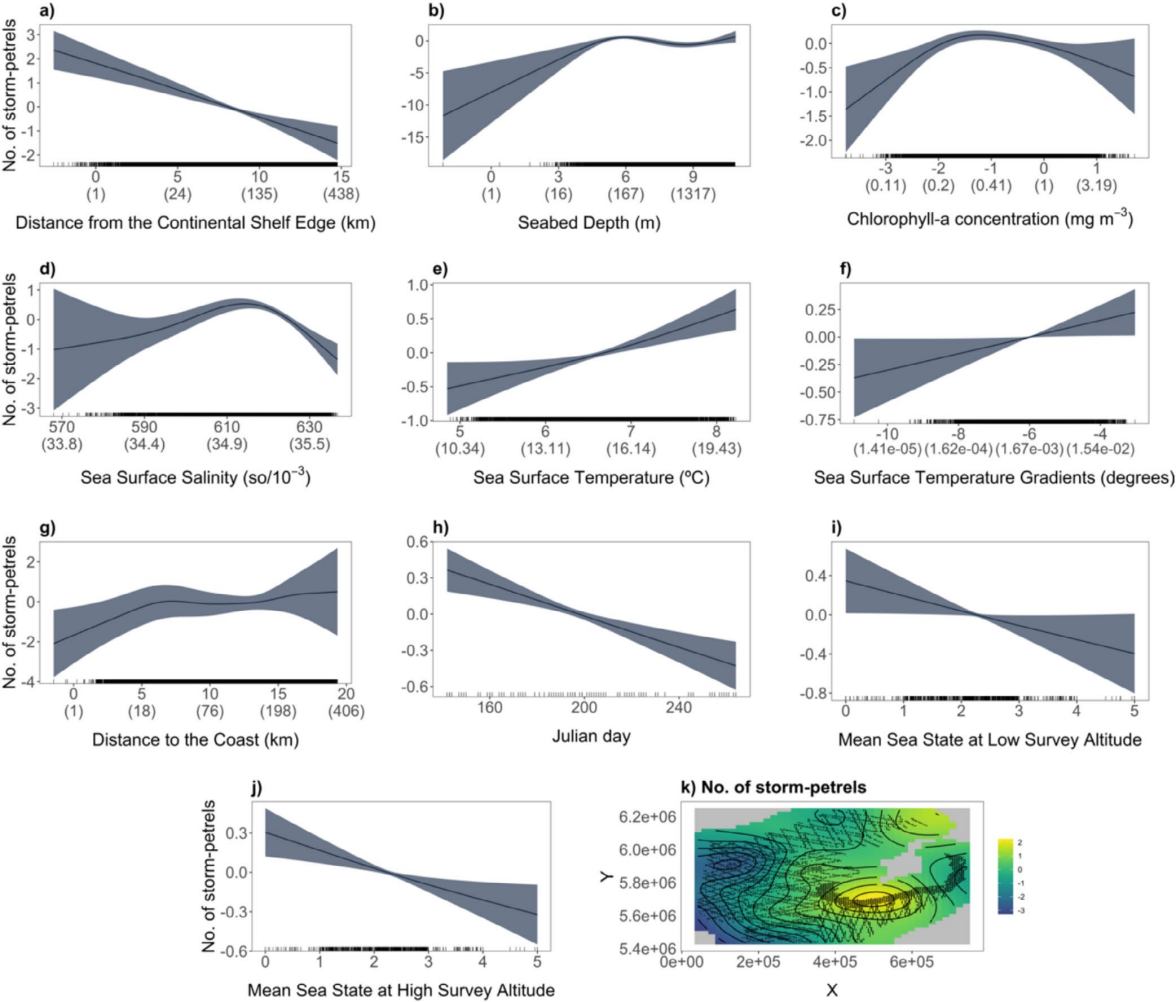
Significant covariates explaining the distribution of storm‐petrels plotted on the link scale. Shaded areas represent the standard error. For transformed variables, the *x*‐axis labels show the transformed scale, followed by corresponding values on the original scale in brackets for easier interpretation.

The spatial smoother of X and Y coordinates exhibited high concurvity with distance to the coast, colony proximity score, distance to the shelf edge, seabed depth and SSS. After testing all combinations of these covariates, the model with the lowest AIC retained all terms except the colony proximity score. In this model, the effect of year was non‐significant, and so it was removed to simplify the predictions. In the final GAM, the effects of chl‐*a* gradient were reduced to zero, whereas seabed slope and SSS gradient were retained but found to be non‐significant (Table 1). This model explained 39.8% of deviance, and the effect of each significant covariate is shown in Figure 2.

**TABLE 1:** GAM model terms explaining the abundance of storm‐petrels per grid cell. Covariates are reported with estimated degrees of freedom (edf).

Model term	df/edf	*X* ^2^ value	*p*
s(x, y)	21.740	247.344	< 0.001
s(sea surface salinity)	3.270	53.928	< 0.001
s(seabed depth)	3.544	42.303	< 0.001
s(distance to continental shelf edge)	1.257	35.980	< 0.001
survey altitude	1	30.330	< 0.001
s(distance to coast)	4.005	22.287	< 0.001
s(Julian day)	1.072	18.500	< 0.001
s(sea surface temperature)	1.304	17.779	< 0.001
s(chlorophyll *a* concentration)	2.758	17.416	< 0.001
s(mean sea state, by = high survey altitude)	0.981	11.165	< 0.001
s(sea surface temperature gradient)	0.819	4.694	0.015
s(mean sea state, by = low survey altitude)	0.796	4.290	0.019
s(sea surface salinity gradient)	0.566	1.526	0.096
s(slope)	0.003	0.004	0.218

**FIGURE 3:**
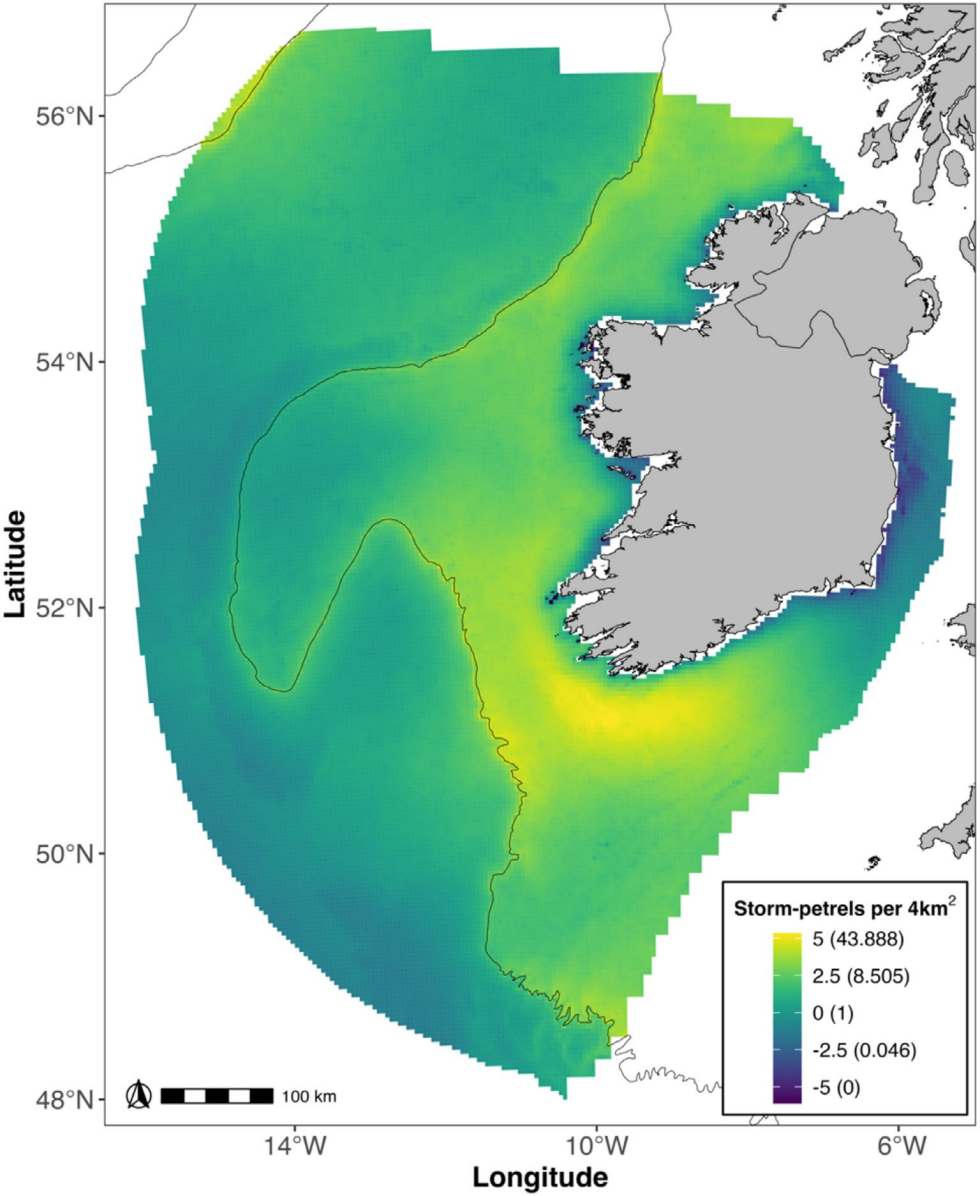
Modelled distribution of storm‐petrels transformed using a Box‐Cox transformation (λ = 0.14). The legend shows the transformed scale followed by the corresponding values on the original scale in brackets for easier interpretation. The black contour line marks the 500 m isobath, indicating the location of the continental shelf edge.

**FIGURE 4:**
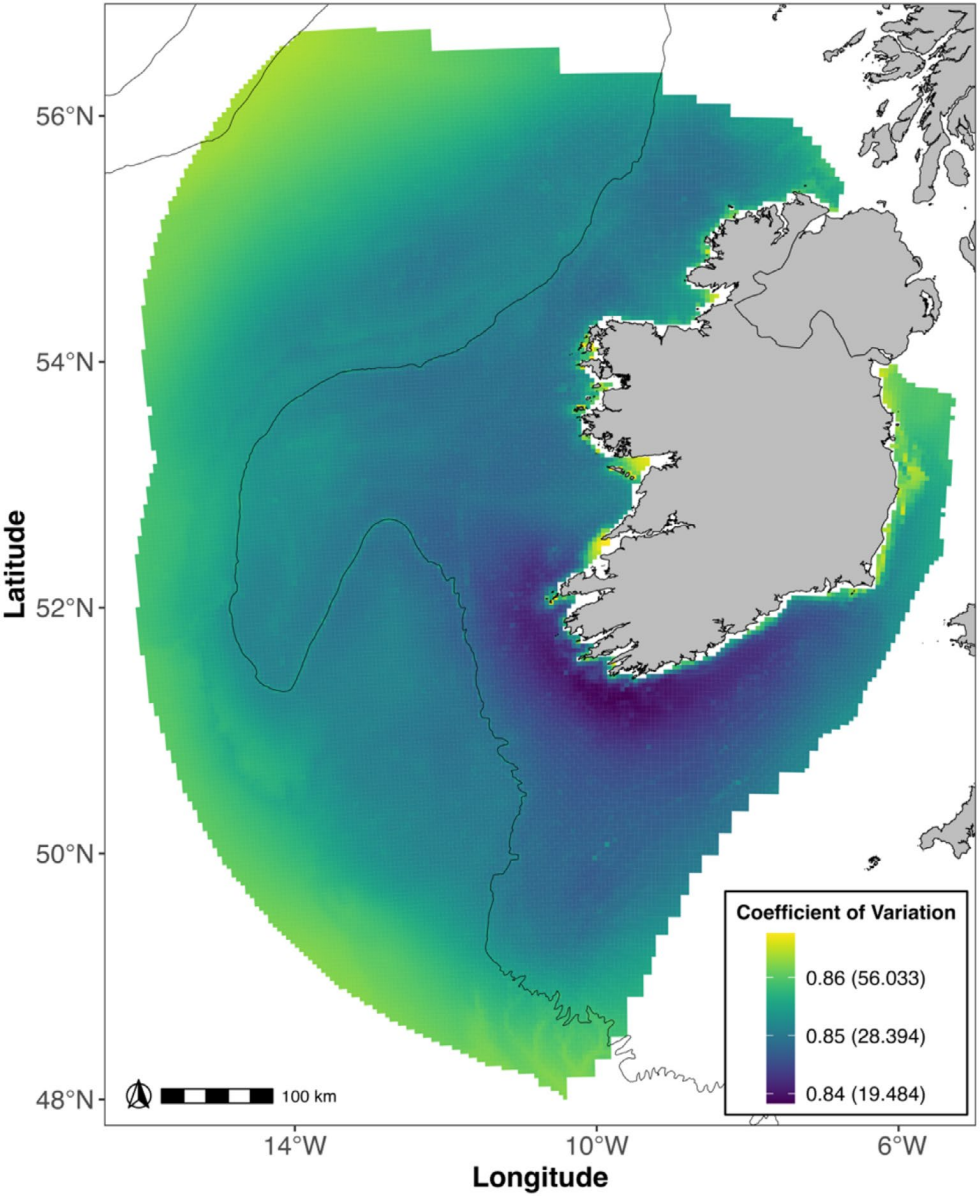
Coefficient of variation per grid cell transformed using a Box‐Cox transformation (λ = −1.15). The legend shows the transformed scale followed by the corresponding values on the original scale in brackets for easier interpretation. Higher values indicate more uncertainty in the prediction. The black contour line marks the 500 m isobath, indicating the location of the continental shelf edge.

Storm‐petrels had a widespread distribution as evident from the extensive sightings of storm‐petrels across the study area (Figure S3) and by the modelled distribution (Figure 3). Storm‐petrels were predicted to occur in high numbers close to the continental shelf edge (Figure 2a) and in waters deeper than 160 m (Figure 2b). Storm‐petrels were found to associate with grid cells with chl‐*a* ranging from 0.2 to 1 mg m^−3^ (Figure 2c), SSS of approximately 35‰ (Figure 2d), high SST (Figure 2e) and high SST gradient values (Figure 2f). Distance to the coast had a strong, curvilinear effect (Figure 2g) and showed that predicted abundance was low at the coast and increased with distance, peaking at 20–40 km with a further peak at ~230 km. Abundance declined throughout the breeding season (Figure 2h), with increasing Beaufort sea state for both survey altitudes (Figure 2i,j), and the XY spline (Figure 2k) accounted for a substantial portion of the variation in the response variable. The model estimated a total abundance of 154,044 (95% CI: 94,347–452,299; Figure S5) storm‐petrels and identified a hotspot of storm‐petrel abundance 20–40 km off the south and south‐west coasts (Figure 3). Prediction uncertainty was greatest in areas with little or no survey effort, especially in the north‐west of the EEZ, and in some coastal areas (Figure 4).

## 4 Discussion

We present the first at‐sea abundance estimate of storm‐petrels for the entire Irish EEZ and greatly improve our understanding of the offshore distribution of the entire population (breeders, failed breeders and non‐breeding immatures, some of which undoubtedly belong to colonies outside of Ireland) during the period of peak storm‐petrel abundance in the North‐East Atlantic.

### 4.1 Detection of Storm‐Petrels Using Aerial Surveys

Aircraft altitude can affect seabird detection by causing disturbances that drive birds away from the transect line, and higher altitudes are expected to make spotting and accurately identifying species more challenging (Camphuysen et al. 2004). As two different survey altitudes were used in this study, it was hypothesised that the lower survey altitude used for fine‐scale transects would result in more sightings of small storm‐petrels. This was confirmed, suggesting that broad‐scale surveys conducted at higher altitudes likely underestimated true storm‐petrel abundance.

The strip‐transect method operates under the assumption that 100% of birds within the designated strip width are accurately identified and detected. Some Leach's storm‐petrels were likely included in the birds detected, but they account for just 0.8% of all storm‐petrels breeding on the island of Ireland (Burnell et al. 2023) and occur primarily off the north‐west coast. We assume that other species misidentification and counting mistakes were not important in this study, since the observers were trained and experienced in the identification of North Atlantic seabirds. Nevertheless, non‐detection errors were likely present in the data. Both models indicated a decline in storm‐petrel numbers with increasing sea state, especially in the higher altitude broad‐scale survey data, suggesting either that storm‐petrels avoid areas with rougher seas or more likely for this species (as wind intensity has not been shown to significantly impact foraging habitat selection; Bolumar Roda et al. 2025), that they remain present but are more difficult to detect under these conditions, resulting in an underestimation of their true abundance. Also, it is expected that storm‐petrels resting on the water were largely undetected as their characteristic flight pattern and white rump plumage, both key features used by observers for detection and identification, are obscured when the bird sits on water with their wings covering their rump. The proportion of time European storm‐petrels spend resting on the water is unknown, making it difficult to control for this potential source of non‐detection error. Further research on the detectability of storm‐petrels during aerial surveys is needed, particularly as aerial surveys are increasingly employed for marine environmental impact assessments and informing conservation efforts (e.g., Ronconi et al. 2022; Virgili et al. 2024). Despite the potential sources of detection bias, the main ones, sea state and survey altitude, are corrected for in our abundance estimates, and one can assume that the patterns of distribution are unlikely to be affected in any significant way.

### 4.2 Abiotic and Biotic Predictors of Distribution

Seabirds are central‐place foragers during the breeding season, meaning that distance to their colony often plays a significant role in shaping their at‐sea distribution (e.g., Skov et al. 2008; Louzao et al. 2012; Chivers et al. 2013). In this study, the colony proximity score, which accounted for both the proximity to and population size of breeding colonies, was excluded from the model due to multicollinearity, suggesting that its influence on storm‐petrel distribution is already captured by other spatial covariates. Moreover, proximity to breeding colonies is expected to be less critical for describing the daytime distribution of storm‐petrels compared to other seabird species because they are highly pelagic and only return to their colonies at night. These birds perform multi‐day foraging trips covering great distances (Rotger et al. 2020; Bolton 2021; De Pascalis et al. 2021), meaning they could be found across a variety of locations within their foraging range during daytime aerial surveys, whereas non‐breeding immatures are not constrained by the need to regularly return to a colony.

Instead, we found that the distance to both the coast and the continental shelf edge was an important variable. European storm‐petrels have been shown to avoid coastal areas during daylight (Pettex, Laran, et al. 2017; Waggitt et al. 2020; Bolton 2021), presumably for predator avoidance or due to insufficient prey availability, a pattern clearly seen in this study. However, GPS data show that usage of coastal areas is prevalent at night as storm‐petrels move to and from their breeding colonies (Bolton 2021) and diet analysis has found evidence of nocturnal coastal foraging by European storm‐petrels during both migration (Thomas et al. 2006) and the breeding season (Albores‐Barajas et al. 2011). This highlights a limitation of visual surveys that rely on daylight hours for observations, as we can only speculate whether the detected daytime hotspots persist, dissipate or expand during the night.

Our results suggest that the importance of the continental shelf edge to storm‐petrels in our study area differs from that seen in other parts of their breeding distribution. Many studies have shown that storm‐petrel density is higher close to the continental shelf edge (e.g., Stone et al. 1995; Pollock et al. 2000; Kober et al. 2010; Arcos et al. 2012; Araújo et al. 2022) and this is to be expected because European storm‐petrels feed on ichthyoplankton, microzooplankton and pelagic fish (D'Elbée and Hémery 1998; Albores‐Barajas et al. 2011), all of which benefit from shelf edge fronts that allow high levels of primary productivity to be sustained (Cox et al. 2018). Although the model results indicated an increase in storm‐petrel abundance near the shelf edge, and the shelf edge is within the species' foraging range (Rotger et al. 2020; Bolton 2021; De Pascalis et al. 2021), the peak in abundance located in the neritic zone suggests that storm‐petrels in our study area found sufficient food closer to the coast and did not need to commute to the shelf edge. This highlights the importance of having region‐specific empirical data when designating areas for the conservation of widely distributed seabirds and not assuming a species' habitat preferences remain consistent across its entire range. This study also underscores the importance of careful consideration when selecting methods for projecting the at‐sea distribution of seabird species for conservation purposes. We note that foraging radius modelling, which is suitable for other species (Grecian et al. 2012; Soanes et al. 2016; Critchley et al. 2020), would not detect our observed European storm‐petrel distribution pattern of coastal avoidance and a distinct, narrow band of high abundance in the neritic zone well within the species' foraging range.

Dynamic environmental variables, such as SST and chl‐*a*, are regularly used as proxies for primary productivity and prey abundance in the marine ecosystem (Tremblay et al. 2009). Foraging areas of many seabird species, including European storm‐petrels, have been linked with low SST and high chl‐*a*, which are characteristic of highly productive upwellings and fronts (e.g., Paiva et al. 2010; Grecian et al. 2016; Domalik et al. 2018; De Pascalis et al. 2021; Bolumar Roda et al. 2025). The waters off the south‐west coast of Ireland are strongly influenced by oceanographic processes that likely result in the region being highly suitable for foraging storm‐petrels during the breeding season. The Irish Shelf Front (ISF) is a haline front that delineates coastal water with a typical salinity of 34.8‰–35.0‰ from more saline oceanic water (Raine 2014). The location of the ISF is defined by the 35.3‰ isohaline (Raine and McMahon 1998) and usually lies 20–40 km offshore (Huang et al. 1991). This aligns with the predicted hotspot of storm‐petrel abundance in the south‐west (Figure 3) and the model's finding that storm‐petrel numbers peak at 35‰ salinity (Figure 2d). Surface chl‐*a* in the region is generally low (0.1–0.6 mg m^−3^; Raine and McMahon 1998), matching the range identified by the model as associated with higher storm‐petrel numbers (Figure 2c). Our results showed a higher abundance of storm‐petrels in warmer waters (Figure 2e), which contrasts with the known preference for foraging in areas of low SST (De Pascalis et al. 2021; Bolumar Roda et al. 2025). However, our model revealed that storm‐petrel numbers were higher in regions associated with steep SST gradients (Figure 2f). SST gradients act as a proxy for sub‐mesoscale thermal oceanographic processes, such as thermal coastal currents, upwellings and eddies, all of which are found in the waters off the Irish south and south‐west coasts during summer months (Raine et al. 1990; Huang et al. 1991; Raine and McMahon 1998; Fernand et al. 2006; Raine 2014), and strongly influence the spatial and temporal distribution of the planktonic community in the region. Coastal currents, in particular, play an important role in transporting nutrients and plankton (Hill et al. 2008), likely resulting in abundant and reliably available prey for storm‐petrels in this area during the breeding season. This result suggests that storm‐petrels are actively targeting the highly productive waters associated with these oceanographic processes for foraging, a behaviour also observed in other regions (De Pascalis et al. 2021; Bolumar Roda et al. 2025).

### 4.3 Storm‐Petrel Abundance Estimates

Our results showed a decline in storm‐petrel abundance over the course of the breeding season (Figure 2h). This pattern largely aligns with expectations as both members of a breeding pair spend the day at sea prior to laying their egg, resulting in high at‐sea abundance, and once incubation begins, one member remains at the nest (Davis 1957), reducing the number of individuals at sea available for detection by aerial surveys. However, an increase in numbers was expected later in the breeding season when the chick is left alone at the nest with both adults foraging at sea. Additionally, a rise in non‐breeding individuals in Irish waters during the second half of the breeding season would contribute to higher at‐sea abundance (Harris et al. 1993; Fowler and Hounsome 1998). Therefore, the continued decline in storm‐petrel numbers may reflect undetected factors, such as reduced detectability later in the season or shifts in distribution resulting in birds being located outside the surveyed areas.

The most recent colony estimates of storm‐petrels breeding in Ireland are 108,423 (95% CI: 91,869–127,085) pairs of European storm‐petrels and 862 (95% CI: 563–1623) pairs of Leach's storm‐petrel (Burnell et al. 2023). Although our surveys likely under‐detected storm‐petrels, as previously discussed, and given that one member of each breeding pair remains at the nest for much of the first half of the breeding season (Davis 1957), our estimate of 154,044 (95% CI: 94,347–452,299) individuals at sea at the median Julian day appears to reflect a reasonable portion of the breeding population. The number of non‐breeding birds included in our data is uncertain, but previous efforts to quantify the proportion of non‐breeding immatures using mist nets and capture‐mark‐recapture methods in the Mediterranean suggest it could range from approximately 0.2–0.5, with results varying between sites and study designs (Sanz‐Aguilar et al. 2010). It is also unclear what proportion of the birds we detected were breeding in other countries, but this could be significant off the north coast of Ireland near some of the larger Scottish colonies. Nevertheless, despite the potential contributions of immatures and birds breeding elsewhere, our modelled distribution is likely representative of the storm‐petrel population breeding around the Irish coastline.

The value of at‐sea surveys for monitoring seabird abundance in coastal and offshore waters is also worth consideration. The importance of colony censuses is well established, especially for local management effort and population monitoring, and yet only a few European storm‐petrel colonies in Britain and Ireland are monitored regularly. Moreover, the highly labour‐intensive national censuses for this species are conducted just once every 10–15 years (Mitchell et al. 2004; Burnell et al. 2023). Until the most recent census (Burnell et al. 2023), the accuracy of most colony counts was unclear because burrow distribution in colonies is patchy, sampling effort was unspecified, and there are several stages where uncertainty is likely introduced into estimates (Brown 2006; Soanes et al. 2012; Arneill 2018). We suggest that more regular at‐sea fine‐scale surveys targeting key hotspots could complement traditional colony censuses. Additionally, estimating the at‐sea abundance of storm‐petrels using aerial surveys can provide essential data for mass‐balanced ecosystem models helping to assess future ecosystem responses to climate change and other pressures (e.g., Ainsworth et al. 2011).

## 5 Conclusion

Observer‐based aerial surveys are an effective way of collecting data on the distribution and abundance of storm‐petrels at sea. However, aircraft altitude and sea state appear to impact the detection of storm‐petrels, and further research into the optimum aerial survey design for this species is warranted. Although the continental shelf edge is identified as an important feature in the distribution of storm‐petrels, and daytime avoidance of coastal waters is clear, the highly productive waters 20–40 km off Ireland's south and south‐west coasts serve as the primary hotspot for storm‐petrel abundance in Irish waters, shaped by a haline shelf front, coastal currents, upwellings and eddies. Since Ireland holds more than 20% of the entire breeding population of the European storm‐petrel, this finding has global significance.

## Author Contributions

**Darren Wilkinson:** conceptualization (equal), data curation (equal), formal analysis (lead), funding acquisition (equal), investigation (equal), methodology (equal), validation (lead), writing – original draft (lead), writing – review and editing (equal). **Jamie Darby:** formal analysis (supporting), writing – review and editing (supporting). **Ashley Bennison:** data curation (equal), investigation (supporting), writing – review and editing (supporting). **Hélder Araújo:** investigation (supporting), writing – review and editing (supporting). **Oriol Giralt Paradell:** data curation (equal), investigation (supporting), validation (supporting), writing – review and editing (supporting). **T. David Tierney:** funding acquisition (equal), methodology (equal), writing – review and editing (supporting). **Emer Rogan:** funding acquisition (equal), investigation (supporting), methodology (equal), project administration (equal), resources (equal), validation (supporting), writing – review and editing (supporting). **John L. Quinn:** conceptualization (equal), formal analysis (supporting), funding acquisition (equal), investigation (equal), methodology (equal), resources (equal), supervision (lead), validation (equal), writing – original draft (supporting), writing – review and editing (equal). **Mark Jessopp:** conceptualization (equal), data curation (equal), formal analysis (supporting), funding acquisition (equal), investigation (equal), methodology (equal), project administration (equal), resources (equal), supervision (supporting), validation (supporting), writing – original draft (supporting), writing – review and editing (equal).

## Conflicts of Interest

The authors declare no conflicts of interest.

## Supporting information


Data S1.

## Data Availability

The data and R scripts that support the findings of this study are openly available in the Dryad Digital Repository at https://doi.org/10.5061/dryad.c2fqz61kc.
