# Supplementary material for: Spatial Modelling of Aerial Survey Data Reveals an Important European Storm‐Petrel Hotspot and Its Underlying Drivers Within the North‐East Atlantic
Source: Ecol Evol. 2025 Jun 30;15(7):e71438. doi: 10.1002/ece3.71438 (PMC12207751; doi:10.1002/ece3.71438)
Supplement: Supplementary file 1 — Data S1. [file ECE3-15-e71438-s001.pdf]

Spatial modelling of aerial survey data reveals an important European storm-petrel hotspot and its underlying drivers within the North-East Atlantic

Darren Wilkinson, Jamie Darby, Ashley Bennison, Hélder Araújo, Oriol Giralt Paradell, T. David Tierney, Emer Rogan, John L. Quinn and Mark Jessopp

Supporting Information

Table S1: Start and end dates for each survey as well as survey type (broad- or fine-scale), areas surveyed, total km^2 surveyed, and percentage of effort conducted in Beaufort sea state ≤ 3. A detailed breakdown of survey dates can be found in Table S6.

Survey Period	Survey Type	Survey start date	Survey end date	No. of survey days	Location	Area surveyed (km^2)	% in Sea State ≤ 3
Summer 2015	Broad	08/06/2015	15/07/2015	14	Strata 1-5	3187.50	59.74
Summer 2016	Broad	21/05/2016	07/07/2016	13	Strata 1-5	3377.83	98.73
Summer 2016	Fine	18/06/2016	05/07/2016	4	East	847.10	98.59
Autumn 2016	Fine	15/09/2016	20/09/2016	4	East	841.86	100
Summer 2021	Broad	07/07/2021	11/09/2021	20	Strata 1-8	4042.95	95.50
Summer 2021	Fine	18/06/2021	21/07/2021	6	South	1036.97	100
Autumn 2021	Fine	14/09/2021	15/09/2021	2	South	473.93	100
Summer 2022	Broad	30/06/2022	14/08/2022	21	Strata 1-8	3695.23	99.94
Summer 2022	Fine	21/06/2022	27/07/2022	7	South-West	1176.40	100
Autumn 2022	Fine	03/09/2022	18/09/2022	6	South-West	1201.01	100

Table S2: λ (lambda) values used in Box-Cox transformation of variables prior to model fitting.

Variable	λ (model 1)	λ (model 2)
Distance to the coast	0.586	0.343
Distance to the continental shelf edge	0.788	0.263
Colony proximity score	-0.343	-0.505
Seabed depth	0.707	0.061
Seabed slope	0.303	-0.061
Chl-*a* concentration	0.020	-0.263
SST	0.909	0.586
SSS	2.000	2.000
Chl-*a* gradient	0.061	-0.020
SST gradient	-0.020	0.020
SSS gradient	0.182	0.061

Table S3: The number of storm-petrel sightings and the total number of storm-petrels observed during each survey. A breakdown of sightings in the grid cells surveyed by both broad- and fine-scale transects can be found in Table S5.

Survey	No. of storm-petrel sightings	Total number of storm-petrels recorded
Summer broad-scale 2015	233	310
Summer broad-scale 2016	337	489
Summer fine-scale 2016	1	1
Autumn fine-scale 2016	7	9
Summer broad-scale 2021	287	528
Summer fine-scale 2021	261	335
Autumn fine-scale 2021	85	175
Summer broad-scale 2022	405	625
Summer fine-scale 2022	767	1266
Autumn fine-scale 2022	359	749

Table S4: GAM model terms explaining the abundance of storm-petrels per grid cell surveyed by both broad- and fine-scale transects. Covariates included as splines are denoted as s(covariate). Terms are reported with either their degrees of freedom (df) or estimated degrees of freedom (edf) depending on whether they are included as parametric terms or splines. Plots of the covariates can be found in Figure S6.

**Model Term**	**df/edf**	X^2 **value**	**p-value**
s(x, y)	16.390	156.033	<0.001
s(seabed depth)	2.512	27.839	<0.001
s(mean sea state, by = high survey altitude)	2.560	27.660	<0.001
year	3	26.059	<0.001
s(Julian day)	0.948	14.718	<0.001
survey altitude	1	5.104	0.024
s(sea surface temperature gradient)	0.815	4.417	0.018
s(sea surface salinity gradient)	0.657	2.051	0.071
s(mean sea state, by = low survey altitude)	0.635	2.031	0.070
s(slope)	0.318	0.530	0.176

Table S5: Total number of storm-petrels observed in the grid cells that were surveyed by both broad- and fine-scale transects (n = 838, Figure S7), and the total survey effort performed by each survey type in these grid cells.

**Survey Type**	**Total number of storm-petrels recorded**	**Survey Effort**
Broad-scale	404	1519 km^2
Fine-scale	1086	2542 km^2

Table S6. Dates on which broad-scale, fine-scale, or both types of survey were completed.

Date	2015	2016	2021	2022
21 May		Broad		
22 May		Broad		
23 May		Broad		
…				
26 May		Broad		
27 May		Broad		
28 May		Broad		
29 May		Broad		
…				
3 June		Broad		
4 June		Broad		
…				
7 June		Broad		
8 June	Broad			
9 June	Broad			
10 June	Broad			
…				
13 June	Broad			
14 June	Broad			
15 June	Broad			
…				
18 June	Broad	Fine	Fine	
19 June			Fine	
…				
21 June				Fine
22 June	Broad		Fine	Fine
23 June	Broad	Fine		Fine
24 June			Fine	
…				
27 June		Broad		
…				
30 June				Broad
…				
2 July			Fine	
3 July		Fine		
…				
5 July		Broad + Fine		Broad
…				
7 July		Broad	Broad	Broad
8 July			Broad	Broad + Fine
9 July	Broad			
10 July			Broad	Fine
…				
12 July	Broad			
13 July	Broad			

Table S6. Continued

Date	2015	2016	2021	2022
14 July	Broad			
15 July	Broad			
…				
17 July			Broad	Broad
18 July				Broad
…				
20 July				Broad
21 July			Broad + Fine	Broad + Fine
22 July			Broad	
23 July			Broad	
24 July			Broad	
25 July			Broad	
26 July				Broad
27 July				Broad + Fine
28 July				Broad
…				
2 August			Broad	
3 August			Broad	Broad
…				
5 August				Broad
6 August				Broad
7 August				Broad
8 August				Broad
9 August			Broad	Broad
…				
11 August				Broad
12 August				Broad
13 August				Broad
14 August				Broad
…				
18 August			Broad	
19 August			Broad	
…				
22 August			Broad	
…				
1 September			Broad	
2 September			Broad	
3 September			Broad	Fine
…				
10 September			Broad	Fine
11 September			Broad	
12 September				Fine
…				
14 September			Fine	
15 September		Fine	Fine	

Table S6. Continued

Date	2015	2016	2021	2022
16 September				Fine
17 September		Fine		Fine
18 September				Fine
19 September		Fine		
20 September		Fine		

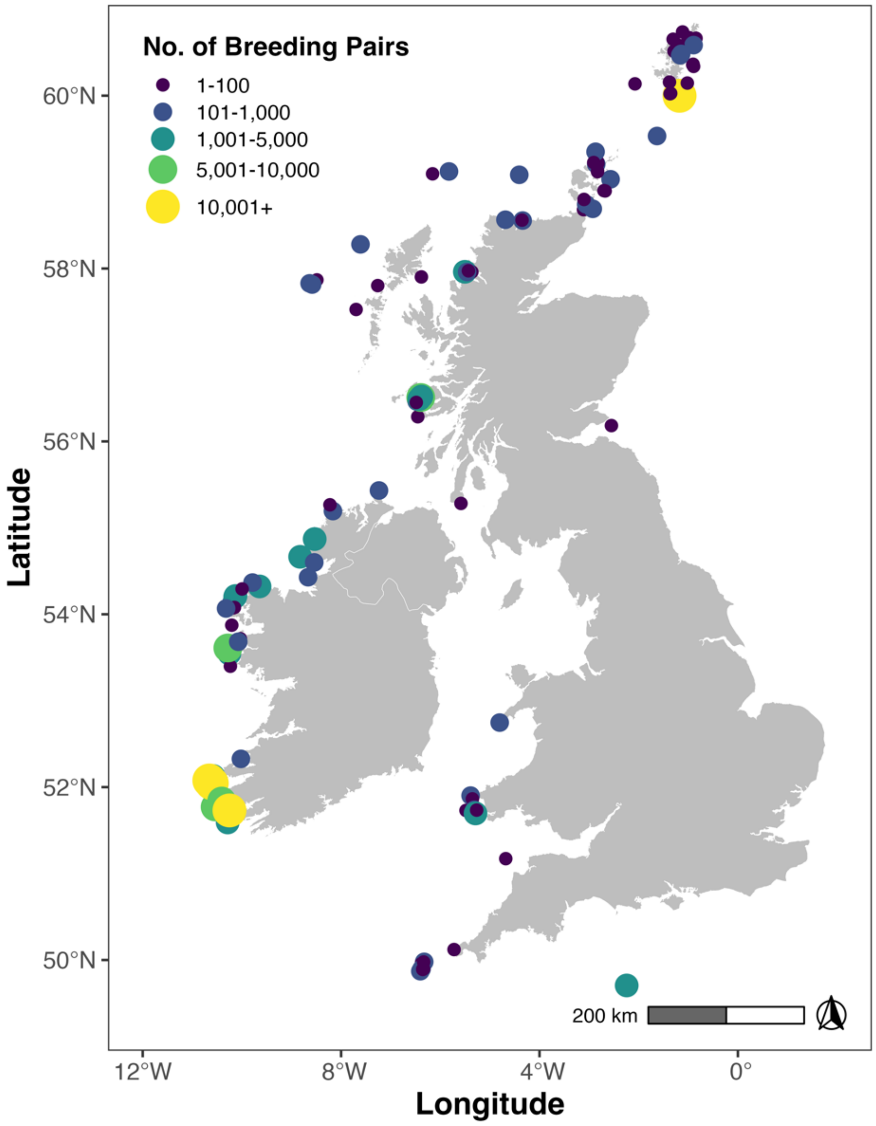

Figure S1: Location of storm-petrel breeding colonies in Ireland and Britain (Burnell et al., 2023) with the size and colour of the symbol representing the number of breeding pairs.

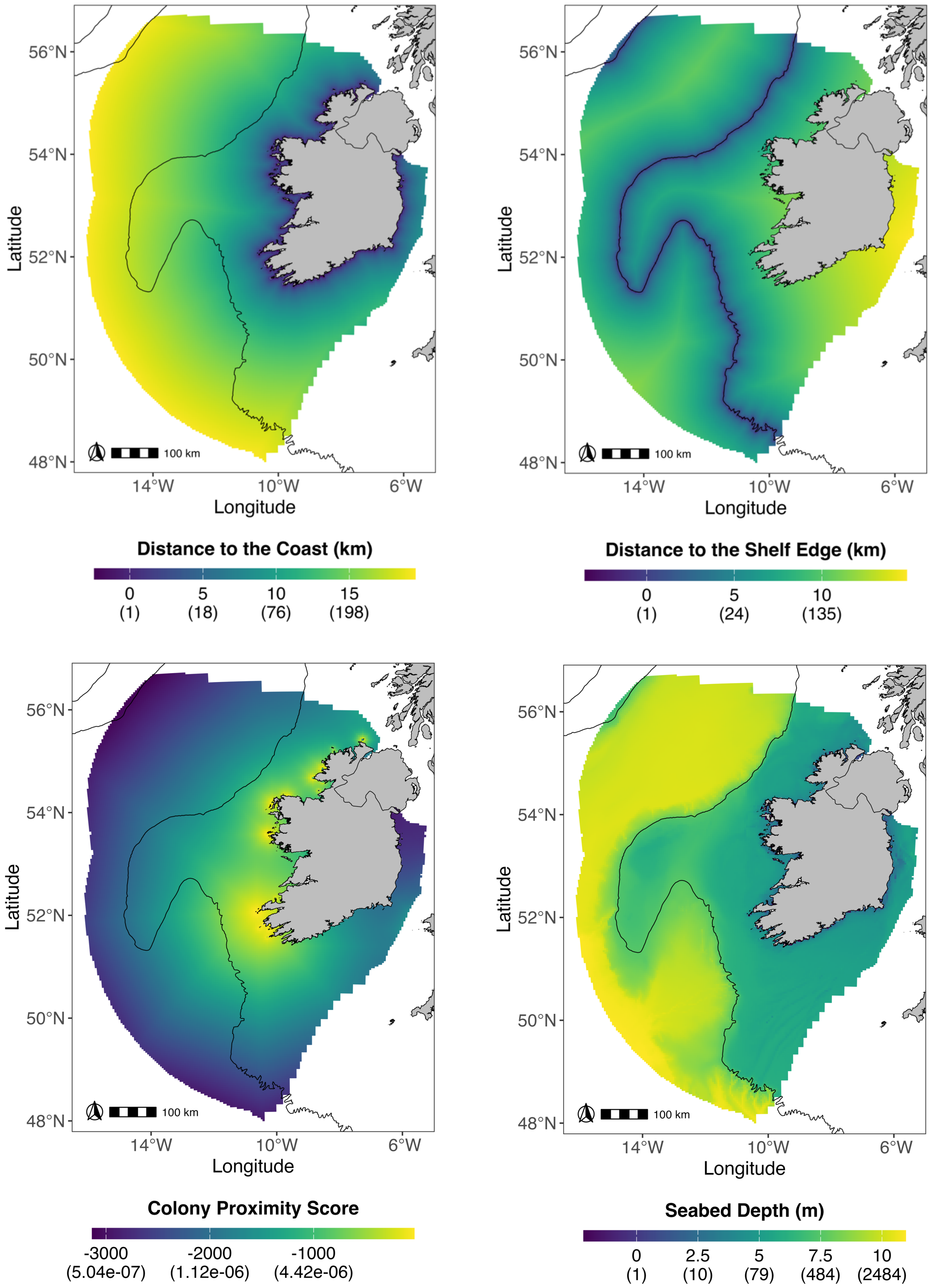

Figure S2: Variables used for distribution predictions. The legends show the transformed scale followed by the corresponding values on the original scale in brackets for easier interpretation. The black contour line marks the 500m isobath, indicating the location of the continental shelf edge.

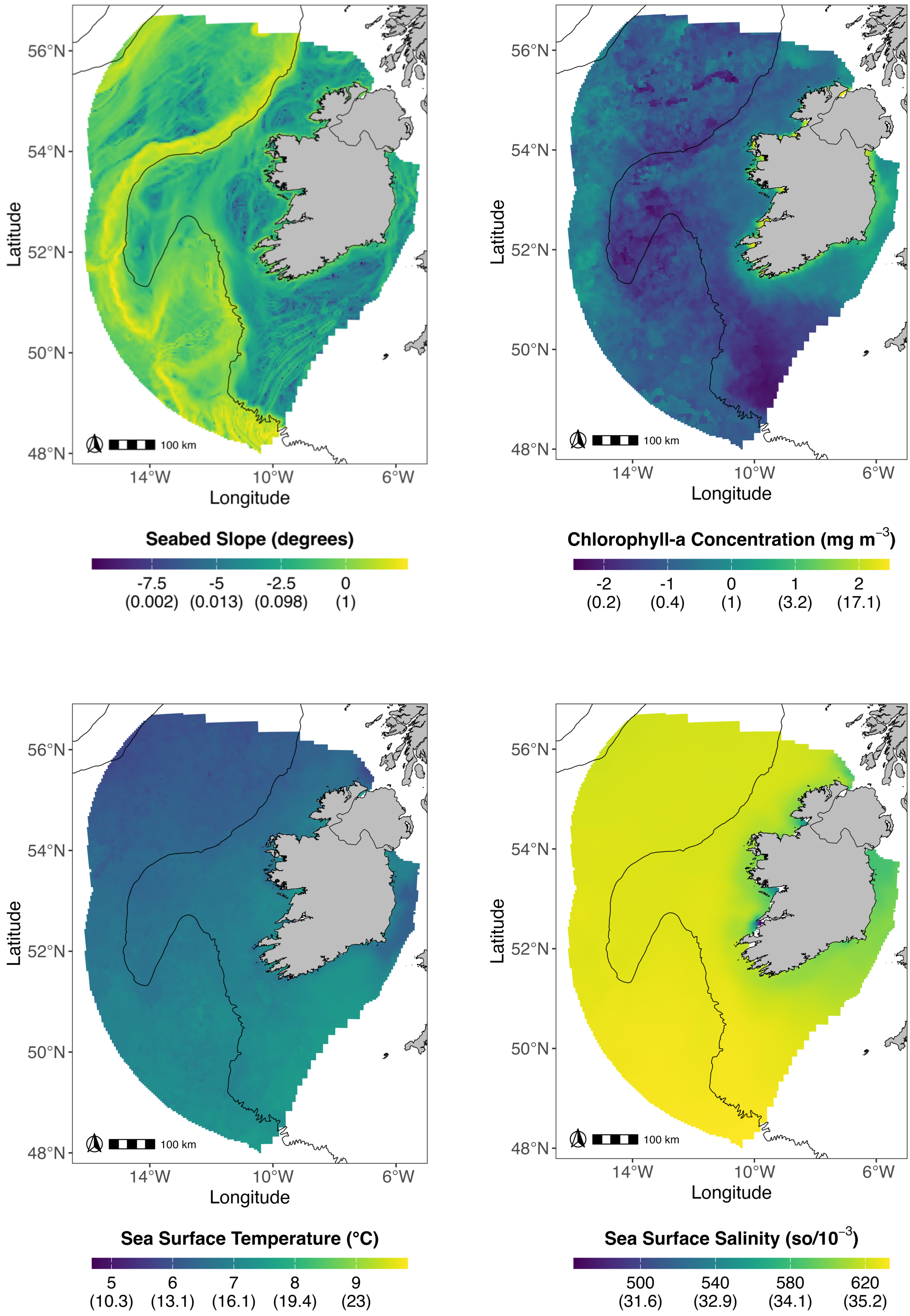

Figure S2: continued

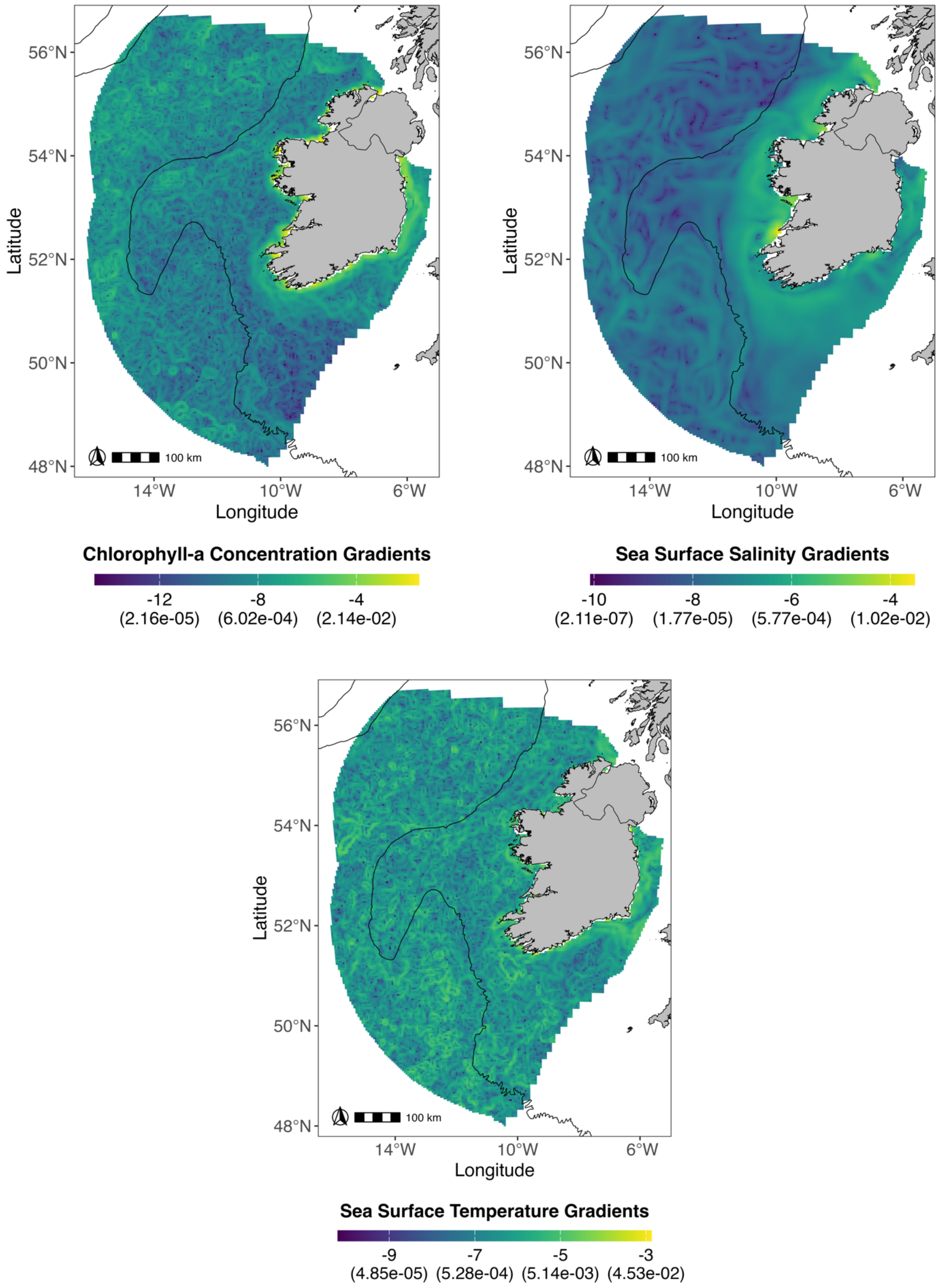

Figure S2: continued

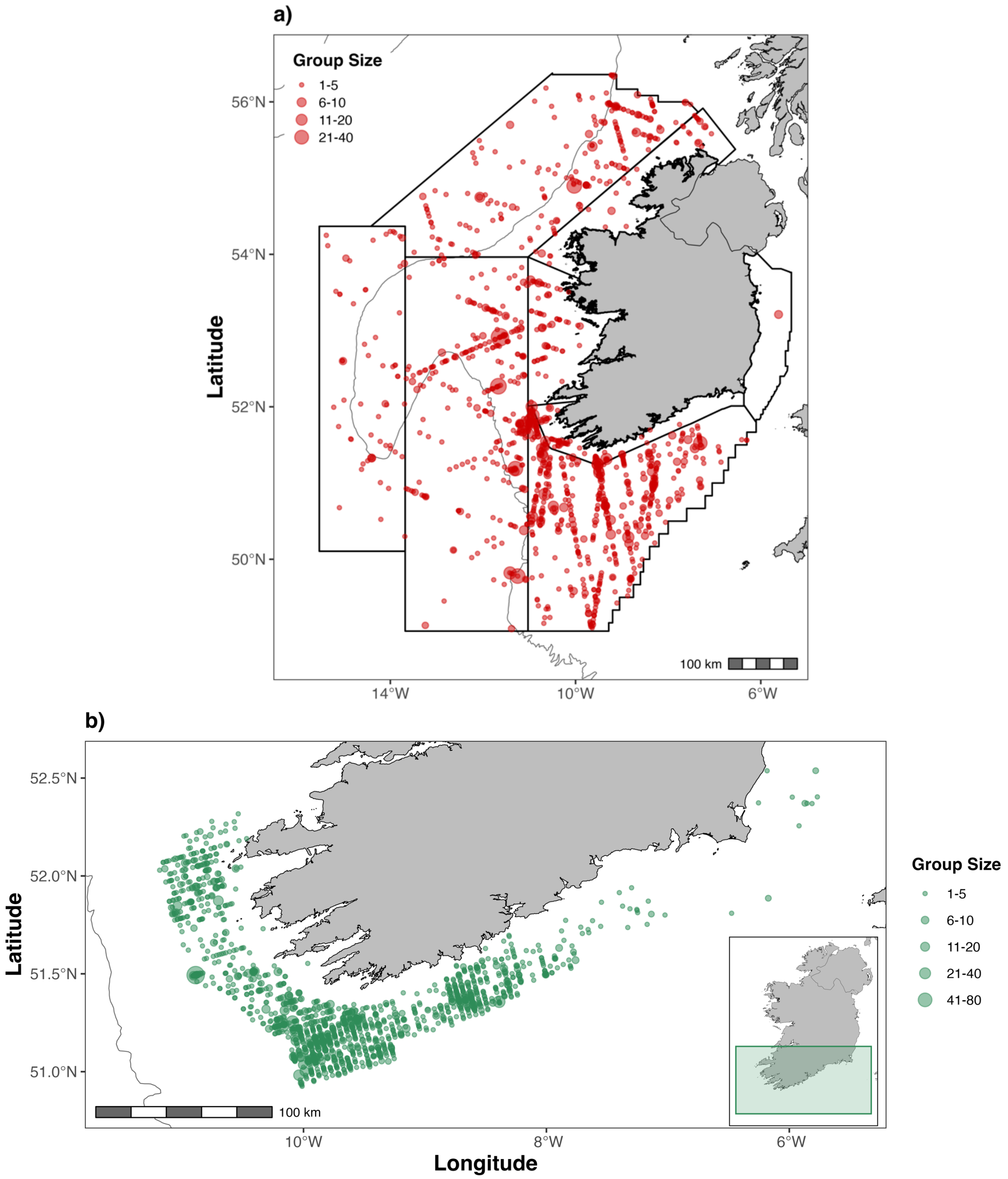

Figure S3: Storm-petrel sightings recorded during all the (a) broad-scale and (b) fine-scale surveys. The size of the point represents storm-petrel group size. The black contour line marks the 500m isobath, indicating the location of the continental shelf edge.

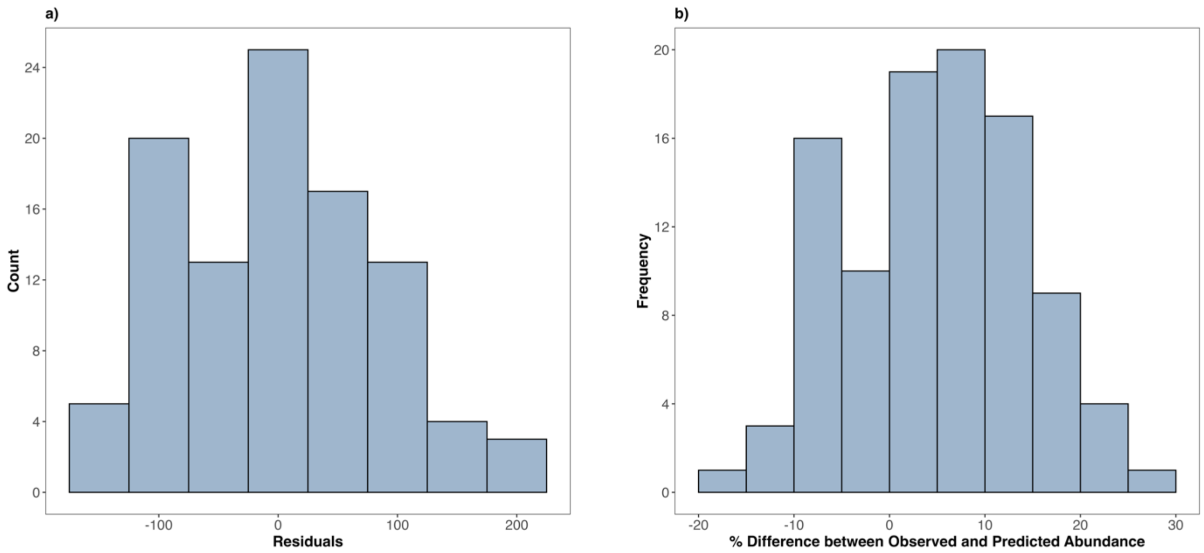

Figure S4: (a) Histogram of the residuals from model validation. (b) Percentage difference between the observed and predicted abundance of the 100 test datasets. Positive values indicate the observed was greater than predicted.

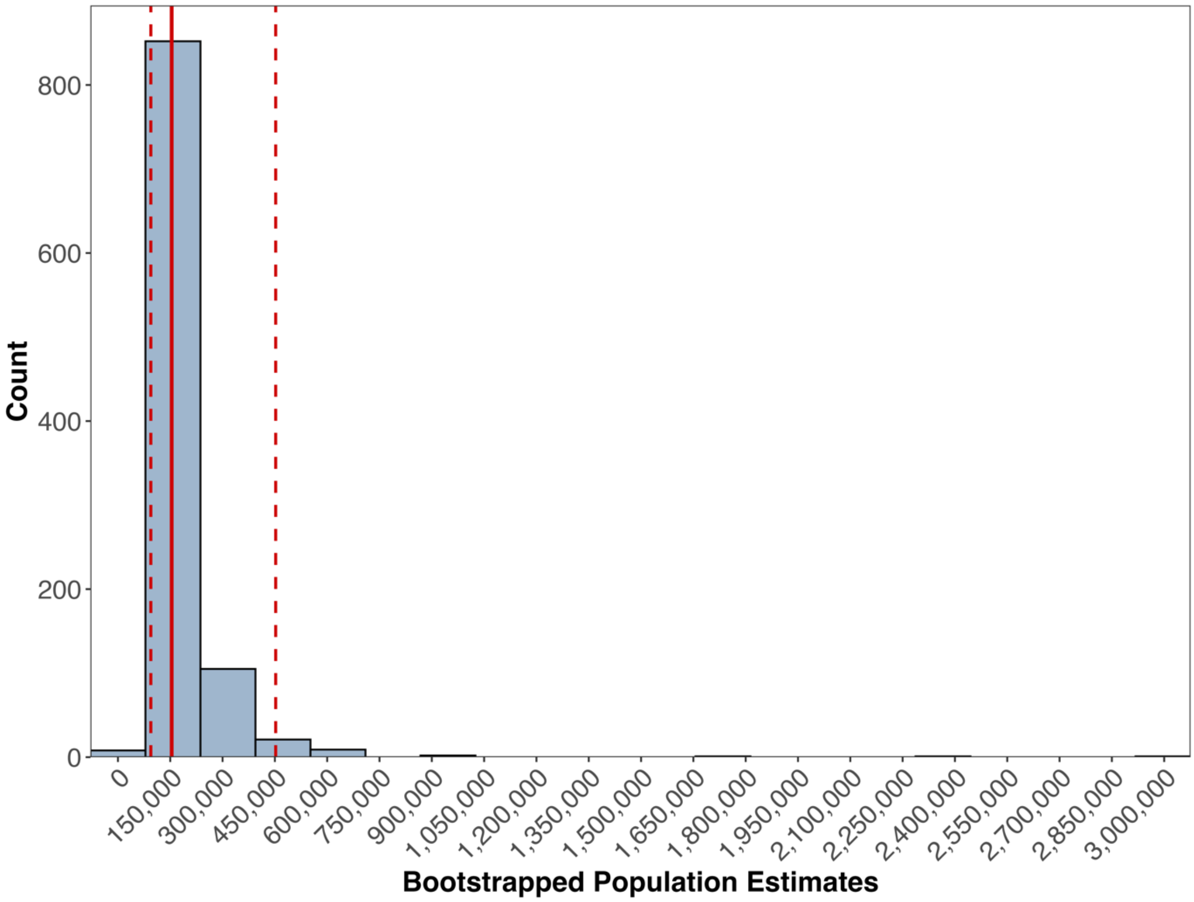

Figure S5. Distribution of population estimates produced by the bootstrapping process. The solid red line marks the population estimate (150,044) and the dashed lines mark the lower and upper 95% confidence intervals (94,347 – 452,299).

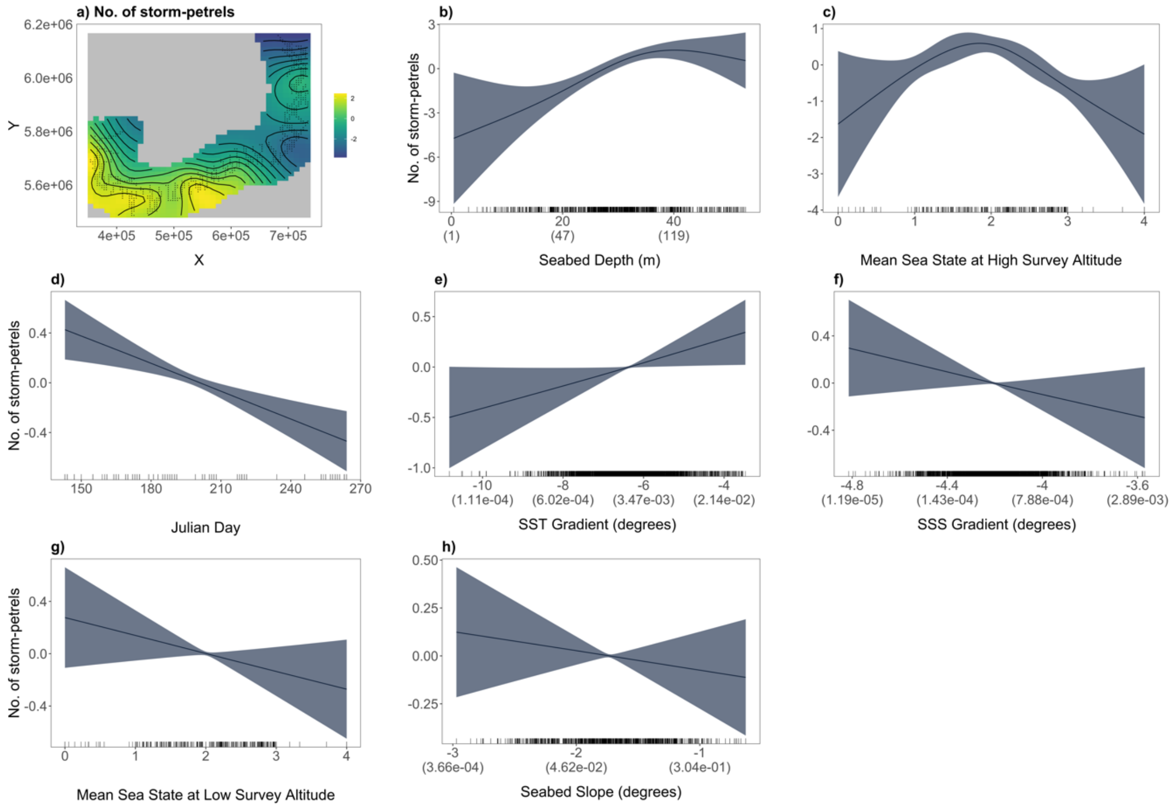

Figure S6: Covariates retained in the model explaining the abundance of storm-petrels per grid cell surveyed by both fine- and broad-scale transects as reported in Table S4 and plotted on the link scale. Shaded areas represent the standard error. For transformed variables, the x-axis labels show the transformed scale, followed by corresponding values on the original scale in brackets for easier interpretation.

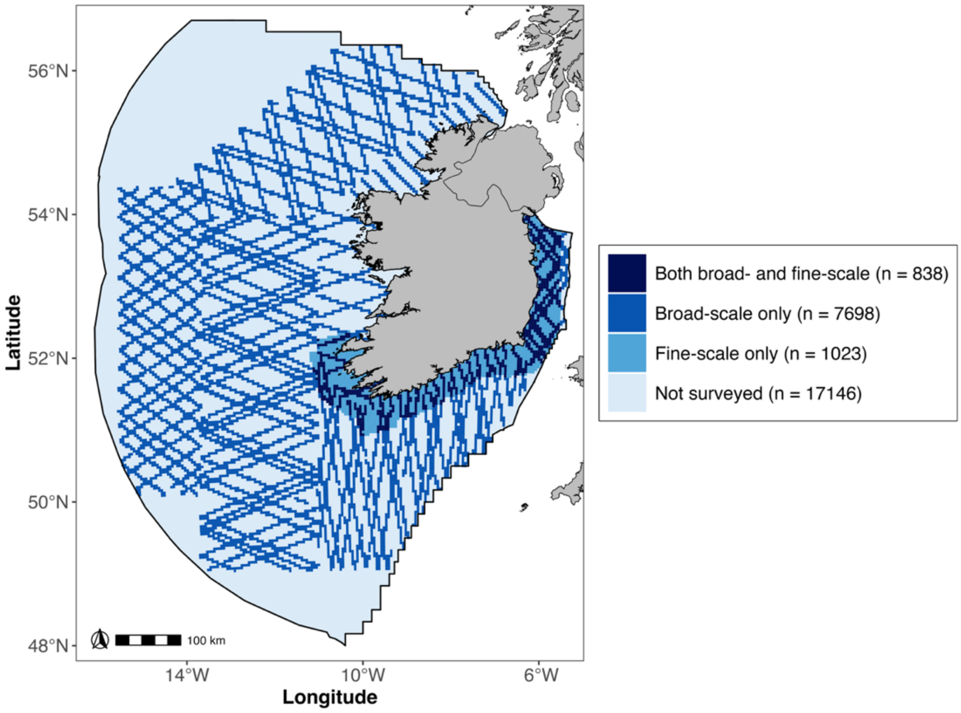

Figure S7: Grid cells surveyed by broad- and fine-scale transects.
